# Association of medically assisted reproduction with offspring cord blood DNA methylation across cohorts

**DOI:** 10.1093/humrep/deab137

**Published:** 2021-06-17

**Authors:** Doretta Caramaschi, James Jungius, Christian M Page, Boris Novakovic, Richard Saffery, Jane Halliday, Sharon Lewis, Maria C Magnus, Stephanie J London, Siri E Håberg, Caroline L Relton, Deborah A Lawlor, Hannah R Elliott

**Affiliations:** MRC Integrative Epidemiology Unit, University of Bristol, Bristol, UK; Population Health Science, Bristol Medical School, University of Bristol, Bristol, UK; MRC Integrative Epidemiology Unit, University of Bristol, Bristol, UK; Population Health Science, Bristol Medical School, University of Bristol, Bristol, UK; Division for Research Support, Oslo Centre for Biostatistics and Epidemiology, Oslo University Hospital, Oslo, Norway; Centre for Fertility and Health, Norwegian Institute of Health, Oslo, Norway; Murdoch Children’s Research Institute, Royal Children’s Hospital, Parkville, VIC, Australia; Department of Paediatrics, University of Melbourne, Parkville, VIC, Australia; Murdoch Children’s Research Institute, Royal Children’s Hospital, Parkville, VIC, Australia; Department of Paediatrics, University of Melbourne, Parkville, VIC, Australia; Murdoch Children’s Research Institute, Royal Children’s Hospital, Parkville, VIC, Australia; Department of Paediatrics, University of Melbourne, Parkville, VIC, Australia; Murdoch Children’s Research Institute, Royal Children’s Hospital, Parkville, VIC, Australia; Department of Paediatrics, University of Melbourne, Parkville, VIC, Australia; MRC Integrative Epidemiology Unit, University of Bristol, Bristol, UK; Population Health Science, Bristol Medical School, University of Bristol, Bristol, UK; Centre for Fertility and Health, Norwegian Institute of Health, Oslo, Norway; Department of Health and Human Services, National Institute of Environmental Health Sciences, National Institutes of Health, Research Triangle Park, NC, USA; Centre for Fertility and Health, Norwegian Institute of Health, Oslo, Norway; MRC Integrative Epidemiology Unit, University of Bristol, Bristol, UK; Population Health Science, Bristol Medical School, University of Bristol, Bristol, UK; MRC Integrative Epidemiology Unit, University of Bristol, Bristol, UK; Population Health Science, Bristol Medical School, University of Bristol, Bristol, UK; Bristol NIHR Biomedical Research Centre, University Hospitals Bristol and Weston NHS Foundation Trust, University of Bristol, Bristol, UK; MRC Integrative Epidemiology Unit, University of Bristol, Bristol, UK; Population Health Science, Bristol Medical School, University of Bristol, Bristol, UK

**Keywords:** assisted reproductive technology, medically assisted reproduction, IVF, DNA methylation, epigenetics, ALSPAC, MoBa, CHART

## Abstract

**STUDY QUESTION:**

Is cord blood DNA methylation associated with having been conceived by medically assisted reproduction?

**SUMMARY ANSWER:**

This study does not provide strong evidence of an association of conception by medically assisted reproduction with variation in infant blood cell DNA methylation.

**WHAT IS KNOWN ALREADY:**

Medically assisted reproduction consists of procedures used to help infertile/subfertile couples conceive, including ART. Due to its importance in gene regulation during early development programming, DNA methylation and its perturbations associated with medically assisted reproduction could reveal new insights into the biological effects of assisted reproductive technologies and potential adverse offspring outcomes.

**STUDY DESIGN, SIZE, DURATION:**

We investigated the association of DNA methylation and medically assisted reproduction using a case–control study design (N = 205 medically assisted reproduction cases and N = 2439 naturally conceived controls in discovery cohorts; N = 149 ART cases and N = 58 non-ART controls in replication cohort).

**PARTICIPANTS/MATERIALS, SETTINGS, METHODS:**

We assessed the association between medically assisted reproduction and DNA methylation at birth in cord blood (205 medically assisted conceptions and 2439 naturally conceived controls) at >450 000 CpG sites across the genome in two sub-samples of the UK Avon Longitudinal Study of Parents and Children (ALSPAC) and two sub-samples of the Norwegian Mother, Father and Child Cohort Study (MoBa) by meta-analysis. We explored replication of findings in the Australian Clinical review of the Health of adults conceived following Assisted Reproductive Technologies (CHART) study (N = 149 ART conceptions and N = 58 controls).

**MAIN RESULTS AND THE ROLE OF CHANCE:**

The ALSPAC and MoBa meta-analysis revealed evidence of association between conception by medically assisted reproduction and DNA methylation (false-discovery-rate-corrected *P*-value < 0.05) at five CpG sites which are annotated to two genes (percentage difference in methylation per CpG, cg24051276: Beta = 0.23 (95% CI 0.15,0.31); cg00012522: Beta = 0.47 (95% CI 0.31, 0.63); cg17855264: Beta = 0.31 (95% CI 0.20, 0.43); cg17132421: Beta = 0.30 (95% CI 0.18, 0.42); cg18529845: Beta = 0.41 (95% CI 0.25, 0.57)). Methylation at three of these sites has been previously linked to cancer, aging, HIV infection and neurological diseases. None of these associations replicated in the CHART cohort. There was evidence of a functional role of medically assisted reproduction-induced hypermethylation at CpG sites located within regulatory regions as shown by putative transcription factor binding and chromatin remodelling.

**LIMITATIONS, REASONS FOR CAUTIONS:**

While insufficient power is likely, heterogeneity in types of medically assisted reproduction procedures and between populations may also contribute. Larger studies might identify replicable variation in DNA methylation at birth due to medically assisted reproduction.

**WIDER IMPLICATIONS OF THE FINDINGS:**

Newborns conceived with medically assisted procedures present with divergent DNA methylation in cord blood white cells. If these associations are true and causal, they might have long-term consequences for offspring health.

**STUDY FUNDING/COMPETING INTERESTS(S):**

This study has been supported by the US National Institute of Health (R01 DK10324), the European Research Council under the European Union’s Seventh Framework Programme (FP7/2007-2013)/ERC Grant agreement no. 669545, European Union’s Horizon 2020 research and innovation programme under Grant agreement no. 733206 (LifeCycle) and the NIHR Biomedical Centre at the University Hospitals Bristol NHS Foundation Trust and the University of Bristol. The UK Medical Research Council and Wellcome (Grant ref: 102215/2/13/2) and the University of Bristol provide core support for ALSPAC. Methylation data in the ALSPAC cohort were generated as part of the UK BBSRC funded (BB/I025751/1 and BB/I025263/1) Accessible Resource for Integrated Epigenomic Studies (ARIES, http://www.ariesepigenomics.org.uk). D.C., J.J., C.L.R. D.A.L and H.R.E. work in a Unit that is supported by the University of Bristol and the UK Medical Research Council (Grant nos. MC_UU_00011/1, MC_UU_00011/5 and MC_UU_00011/6). B.N. is supported by an NHMRC (Australia) Investigator Grant (1173314). ALSPAC GWAS data were generated by Sample Logistics and Genotyping Facilities at Wellcome Sanger Institute and LabCorp (Laboratory Corporation of America) using support from 23andMe. The Norwegian Mother, Father and Child Cohort Study is supported by the Norwegian Ministry of Health and Care Services and the Ministry of Education and Research, NIH/NIEHS (Contract no. N01-ES-75558), NIH/NINDS (Grant nos. (i) UO1 NS 047537-01 and (ii) UO1 NS 047537-06A1). For this work, MoBa 1 and 2 were supported by the Intramural Research Program of the NIH, National Institute of Environmental Health Sciences (Z01-ES-49019) and the Norwegian Research Council/BIOBANK (Grant no. 221097). This work was partly supported by the Research Council of Norway through its Centres of Excellence funding scheme, Project no. 262700.

D.A.L. has received support from national and international government and charity funders, as well as from Roche Diagnostics and Medtronic for research unrelated to this study. The other authors declare no conflicts of interest.

**TRIAL REGISTRATION NUMBER:**

N/A.

## Introduction

The number of children conceived using ART has increased steadily over the last four decades. ART include any treatment that consists of *in vitro* handling of human sperm, oocytes and embryos such as IVF/ICSI and gamete and embryo cryopreservation. More broadly, medically assisted reproduction refers to any treatment/intervention for fertility impairment and infertility, including ART and other procedures such as ovulation induction (OI) and intrauterine insemination that might use hormones to stimulate oocyte production but do not usually handle gametes (Zegers-Hochschild *et al.*, 2017). The timing of developmental epigenetic reprogramming coinciding with fertility interventions and the susceptibility of DNA methylation to environmental stresses have led to suggestions that fertility interventions may alter the offspring’s DNA methylation patterns (Canovas *et al.*, 2017). Alterations in the embryo demethylation and re-methylation processes due to medically assisted reproduction can be caused by nutritional stress due to *in vitro* culture conditions, hormonal overstimulation of the ovary beyond the physiological range due to superovulation-inducing procedures, or a combination of the two exposures (Doherty *et al.*, 2000; Mann *et al.*, 2004; Market-Velker *et al.*, 2010a, 2010b; Market-Velker *et al.*, 2012; White *et al.*, 2015; Velker *et al.*, 2017). Medically assisted reproduction procedures may subject gametes and early embryos to environmental stress which may impact the development of the foetus, for example, by disruptions in the expression of imprinted genes (Sakian *et al.*, 2015). Gamete and embryo cryopreservation methods can also affect DNA methylation (Van Heertum and Weinerman, 2018; Cao *et al.*, 2019).

Earlier studies investigating the association of ART and DNA methylation at birth have focused on imprinted genes and found mixed results which overall point to weak evidence of any association when meta-analyzed (Lazaraviciute *et al.*, 2014). Other studies have included other non-imprinted candidate genes, resulting in the discovery of some associations between treatment and methylation in cord blood and placental cells (Katari *et al.*, 2009; Tierling *et al.*, 2010; Song *et al.*, 2015). More recent studies have moved from a candidate gene to a genome-wide analysis approach. Some genome wide studies have identified differential methylation in neonatal blood cells between ART (or, more specifically, IVF) and controls (Melamed *et al.*, 2015; Estill *et al.*, 2016; Castillo-Fernandez *et al.*, 2017; El Hajj *et al.*, 2017; Novakovic *et al.*, 2019). However, other similar studies have found little evidence (Gentilini *et al.*, 2018; Choufani *et al.*, 2019), perhaps owing to small sample sizes, comparisons with different procedures across some studies and consequently insufficient power to detect true associations. Only one study has included more than 100 conceptions through ART (Novakovic *et al.*, 2019), and, although it found several associations, it did not control for potential confounding from parental characteristics.

Whilst different ART procedures have been associated with a wide range of adverse perinatal and longer-term offspring outcomes (Kallen *et al.*, 2010; Hansen *et al.*, 2013; Chen and Heilbronn, 2017; Elias *et al.*, 2020), a large recent study with follow-up to 22–35 years, did not confirm the associations with longer-term outcomes (Halliday *et al.*, 2019). Moreover, the extent to which any associations with adverse outcomes are related to multiple pregnancies, the effect of underlying causes of infertility, selection bias or confounding is unclear. In relation to adverse perinatal outcomes such as pregnancy loss, preterm birth and small for gestational age, increasing evidence suggests that multiple pregnancies explain much of the increased risk (Nelson and Lawlor, 2011; Lawlor and Nelson, 2012). Recent evidence from the European Surveillance of Congenital Anomalies (EUROCAT) suggests that a substantial proportion of associations with congenital anomalies may be due to underlying causes of infertility rather than ART *per se* (Davies *et al.*, 2012; Levi Setti *et al.*, 2016). However, these and studies of ART with longer-term offspring outcomes often rely on large-scale clinical and administrative datasets with minimal data on potential confounders. The use of longitudinal birth cohorts may be useful for assessing causal effects of ART or, more broadly, medically assisted reproduction on offspring health as they typically collect data on a range of potential confounders. The identification of associations of medically assisted reproduction with differential DNA methylation in genes that have established associations with health outcomes could be used to prioritize offspring disease outcomes for more detailed causal analyses that might be mediated by DNA methylation. Interrogating DNA methylation and its potential role as a mediating mechanism linking ART or, more broadly, medically assisted reproduction to health consequences in the offspring may provide insights into the biological pathways involved.

The aim of this study was to determine the association between having been conceived through medically assisted reproduction and genome-wide variation in cord blood DNA methylation at >450 000 CpG sites using the Illumina Methylation 450K BeadChip across multiple cohorts. Medically assisted reproduction is used here to describe the exposure studied as one of the cohorts included did not provide sufficient information for us to be certain the procedures were ART. Building on previous studies, we have used a multi-cohort approach with meta-analysis and replication, while interrogating the whole genome. We have also used functional analysis methods to attempt to characterize the biological implications of the findings in the context of long-term health outcomes.

## Materials and methods

The overall approach to our methods was to pool results from epigenome-wide association studies (EWAS) in two general population birth cohorts, the Avon Longitudinal Study of Parents and Children (ALSPAC) and the Norwegian Mother, Father, and Child Birth Cohort (MoBa), as discovery analyses. We then explored replication of any discovery associations in an independent study, the ‘Clinical review of the Health of adults conceived following Assisted Reproductive Technologies’ (CHART) cohort.

### Study populations

#### Avon longitudinal study of parents and children (ALSPAC)

ALSPAC is a population birth cohort that enrolled 14 541 pregnancies from residents in and around the city of Bristol in the South West of the UK with expected delivery dates between 1991 and 1992 (Boyd *et al.*, 2013; Fraser *et al.*, 2013). The study website contains details of all the data that is available through a fully searchable data dictionary and variable search tool: http://www.bristol.ac.uk/alspac/researchers/our-data/.

In ALSPAC, a subset of 1018 mother–offspring pairs had DNA methylation measured within the Accessible Resource for Integrated Epigenomics Studies (ARIES) (http://www.ariesepigenomics.org.uk/) (Relton *et al.*, 2015). ALSPAC–ARIES participants were selected based on the availability of DNA samples at two time points for mothers and three time points for the offspring (Relton *et al.*, 2015). After quality control checks and exclusion of multiple pregnancies, N = 857 pairs had reliable cord blood DNA methylation data to include in this study. After removing N = 21 participants without data about method of conception and a further N = 52 participants without all the covariates, we had a sample of N = 784 (77% of the original ARIES samples). In comparison to the overall ALSPAC cohort, the subset of ARIES mother–offspring pairs is slightly older, more likely to have a non-manual occupation and less likely to have smoked during pregnancy (Relton *et al.*, 2015).

A second ALSPAC subset, the ALSPAC-Medically assisted reproduction (ALSPAC-MAR) subsample, also had cord blood DNA methylation measured. This subset consisted of additional ALSPAC participants selected to maximize the number of children conceived through medically assisted reproduction and hence increase power. One hundred seventy-eight infants conceived with medically assisted reproduction procedures, with cord blood samples, and 241 naturally conceived controls were selected for epigenome wide DNA methylation analyses. The 241 naturally conceived participants were not a random sample but consisted of participants selected for other epigenetic experiments that were run in the lab at the same time as the medically assisted reproduction cases (arthritis, diabetes, eating disorder, pre-eclampsia, hypertension, BMI, child autism and child undescended testicle). The distribution of these traits across the ALSPAC-MAR cases and control participants is shown in Supplementary Table SI. After removing those without all the covariates, there were N = 345 participants (82% of the additional subset; N = 155 cases and N = 190 non-controls). In both subsets of ALSPAC, information on the specific procedure used meant that we could not distinguish ART from the broader definition of medically assisted reproduction and hence for this cohort and in general in relation to exposure we use the latter term rather than ART. Written informed consent was obtained from all participants. The protocols and types of chip used for measurement of epigenome wide DNA methylation were the same for both ALSPAC samples (Relton *et al.*, 2015). Ethical approval for the ALSPAC data was granted by the ALSPAC law and ethics committee and local Research Ethics Committees. The details of the ethical approval at each stage of the study can be found in the ALSPAC website: http://www.bristol.ac.uk/alspac/researchers/research-ethics/.

#### The Norwegian Mother and Child Cohort Study

Participants represent two subsets of mother–offspring pairs from the national Norwegian Mother and Child Cohort Study (MoBa), a prospective population-based pregnancy cohort study conducted by the Norwegian Institute of Public Health (Magnus *et al.*, 2006, 2016). The years of birth for MoBa participants ranged from 1999 to 2009 and the cohort includes more than 114 000 children, 95 000 mothers and 75 000 fathers. Two subsets of children also have cord blood DNA methylation available. The two subsets are referred to here as MoBa1 and MoBa2. MoBa1 is a subset of a larger study within MoBa that includes a cohort random sample and newborns who developed asthma by age 3 years (Haberg *et al.*, 2011), of which N = 1068 children had cord blood DNA methylation available after quality control exclusions (Joubert *et al.*, 2012). MoBa2 consists of a further 685 participants who were also a random cohort sample and newborns who developed asthma that was still present at age seven (157 with asthma) (Reese *et al.*, 2019). MoBa2 participants did not overlap with those in MoBa1 and the protocol and type of chips used for DNA methylation analyses were the same in both subsets. Cord blood DNA methylation data after quality control exclusions were available in N = 680 MoBa2 children. MoBa1 and MoBa2 data were pooled together and N = 1518 (22 conceived by ART) of these had data on ART and all the covariates included in the analysis.

Ethical approval for MoBa data was granted by Regional Committee for Ethics in Medical Research of South/East Norway, the Norwegian Data Inspectorate, and the Institutional Review Board of the National Institute of Environmental Health Sciences. Written informed consent was obtained from all MoBa participants.

### Medically assisted reproduction assessment

ALSPAC participants were sent a postal questionnaire at 12 weeks gestation that asked a two-part question: (i) ‘Did you use any treatments to help conceive this pregnancy?’, a binary yes/no response, and (ii) ‘If yes which one?’, a text answer. We used the first question to create a binary variable for the main analysis. We used the text response to the second question to perform a sensitivity analysis where the medically assisted reproduction category included only mothers who responded having used ART (IVF or gamete intrafallopian transfer), IUI, or OI by hormones, whilst excluding those who reported using male infertility treatments, natural monitoring, other fertility treatments, other treatment (not fertility), no treatment or unknown and those with mismatches between the two questions (N = 7 removed in ALSPAC–ARIES and N = 28 removed in ALSPAC-MAR).

In MoBa, ART information was obtained from the medical birth registry. The notification form completed by the midwife attending the delivery indicated whether the pregnancy was conceived by IVF, ICSI or other/unknown ART methods, as a single response.

### DNA methylation

In the ALSPAC-ARIES and ALSPAC-MAR subsamples, DNA was extracted from previously collected and frozen cord blood. DNA was then bisulphite converted using Zymo EZ DNA MethylationTM kit (Zymo, Irvine, CA, USA) and methylation was measured at 485 512 CpG sites using the Illumina Infinium HumanMethylation450K BeadChip assay according to manufacturers’ protocols (Relton *et al.*, 2015). The methylation arrays were scanned with an Illumina iScan and the initial quality was checked with GenomeStudio (version 2011.1). The methylation data pre-processed using *meffil* package (Min *et al.*, 2018). The process included quantile normalization and quality checks such as sex chromosomes mismatches and genotype mismatches.

In MoBa1, umbilical cord blood samples were collected and frozen at birth at −80°C. All biological material was obtained from the Biobank of the MoBa study (Paltiel *et al.*, 2014). Bisulphite conversion was performed using the EZ-96 DNA Methylation kit (Zymo Research Corporation, Irvine, CA, USA) and DNA methylation was measured at 485 577 CpGs in cord blood using the Illumina Infinium HumanMethylation450 BeadChip array (Joubert *et al.*, 2012). Raw intensity (.idat) files were handled in R using the *minfi* package to calculate the methylation level at each CpG as the beta-value and the data were exported for quality control and processing. Similar sample specific quality control was performed in MoBa1 and MoBa2, as follows: control probes (N = 65) and probes on X (N = 11 230) and Y (N = 416) chromosomes were excluded in both datasets. Remaining CpGs missing > 10% of methylation data were also removed (N = 20 in MoBa1, none in MoBa2). Samples indicated by Illumina to have failed or have an average detection *P*-value across all probes < 0.05 (N = 49 MoBa1, N = 35 MoBa2) and samples with gender mismatch (N = 13 MoBa1, N = 8 MoBa2) and GWAS outliers (N = 6 MoBa1, N = 4 MoBa2) were also removed. For MoBa1 and MoBa2, we accounted for the two different probe designs by applying the intra-array normalization strategy Beta Mixture Quantile dilation (BMIQ) (Bibikova *et al*., 2011). After quality control exclusions, the sample sizes of the cord blood DNA methylation data (including with missing conception data and covariates) were 1056 for MoBa1 and 680 for MoBa2. To account for differences between MoBa1 and MoBa2, we also adjusted for the different design variables, such as sample pull or selection factors (MoBa2) with a fixed effect variable.

### EWAS meta-analysis

We estimated the association between medically assisted reproduction and cord blood DNA methylation at all the CpGs that passed QC in ALSPAC–ARIES, ALSPAC-MAR and MoBa by running linear regression models of medically assisted reproduction (exposure) on DNA methylation (outcome) using the *CpGassoc* package in R (Barfield *et al.*, 2012). To control for batch, we included a term for bisulphite treatment plate. We used an algorithm and a reference panel that is suitable for cord blood (Bakulski *et al*., 2016) to calculate cell type composition and added estimated cell counts as covariates. Additionally, we adjusted for infant sex at birth and potential confounding by maternal age at delivery, maternal BMI and maternal smoking during the first trimester of pregnancy. In ALSPAC, maternal pre-pregnancy weight, height and smoking were obtained through self-report in questionnaires completed in the first trimester of pregnancy whereas maternal age at delivery and infant sex were extracted from obstetric records. Smoking during the first trimester was categorized into a binary variable of ‘Did not smoke’ and ‘Smoked’. In MoBa, for both datasets, information maternal pre-pregnancy weight height and smoking was obtained through self-report questionnaires completed at around 17 weeks of gestation; maternal age at delivery and infant sex were obtained from the birth registry records. Smoking was categorized in a three-level variable, ‘non-smoker’, ‘stopped early in pregnancy’ and ‘smoked throughout pregnancy’.

A meta-analysis of the summary statistics of the EWAS results from each study sample (ALSPAC–ARIES, ALSPAC-MAR and MoBa) was carried out using METAL (version 2011-03-25) (Willer *et al.*, 2010), using a fixed effects model with inverse variance weighting (Rice *et al.*, 2018). Since in the MoBa EWAS the X and Y chromosome were omitted, the meta-analysis excluded CpGs on these chromosomes.

### Replication in an independent cohort

We sought replication of results for all CpG sites with *P*_FDR_ <0.05 in the meta-analysis EWAS in an independent study sample, the ‘Clinical review of the Health of adults conceived following Assisted Reproductive Technologies’ (CHART) cohort. CHART is an Australian cohort of N = 547 adults conceived by IVF and N = 549 naturally conceived controls (Lewis *et al.*, 2017). In a subsample of N = 193 ART-conceived and N = 86 non-ART adults who gave their consent for epigenetic analyses, DNA methylation was measured in DNA isolated from neonatal blood spots (Guthrie spots) that were collected at 2–3 days after birth using the Illumina InfiniumMethylationEPIC BeadChips array. Pre-processing was carried out using *MissMethyl* and *minfi* R packages and normalization was performed using SWAN (Maksimovic *et al.*, 2012). Quality checks involved the exclusion of samples with mean detection *P*-value > 0.01, probes with detection *P*-value > 0.01, those associated with SNPs and cross-reactive probes. Cell composition was estimated using the Bakulski cord blood cell reference method (Bakulski *et al.*, 2016). Linear regression modelling was performed using *limma* R package and included sample plate, cell proportions (six cell types), infant sex and maternal age at delivery covariates (data were not available in this study to adjust for potential confounding by maternal pre-pregnancy BMI or pregnancy smoking). After quality control exclusions, there were 146 ART cases and 58 controls with data on ART and covariates to use in this analysis. Ethical approval for the CHART study was granted by the Royal Children’s Hospital Human Research Ethics Committee (RCH HREC Project 33163). All participants for this study gave written informed consent.

### Biological characterisation

CpG sites associated with medically assisted reproduction were compared to those listed in the EWAS catalogue (http://www.ewascatalog.org/) in July 2019. Studies indexed by the EWAS catalogue included at least 100 000 CpG sites in analyses and had sample sizes of at least 100 individuals.

Gene ontology (GO) enrichment (Ashburner *et al.*, 2000; The Gene Ontology Consortium, 2017) for the first 100 CpG sites from the meta-EWAS ranked by ascending *P*-value was conducted using the R package *missMethyl* (Phipson *et al.*, 2016). This method takes into account the differing number of probes per gene found on the array which can otherwise bias results (Geeleher *et al.*, 2013). Since hyper- and hypo-methylation at CpG sites in our analysis were likely to be biologically distinct we stratified our enrichment analysis accordingly.

Enrichment analyses were conducted using the R package LOLA (Sheffield and Bock, 2016). Input was genomic coordinates of the first 100 CpG sites from the meta-EWAS ranked by ascending *P*-value and the background set was the genomic coordinates all array CpG sites included in the meta-EWAS. The LOLA core region set was used to test for enrichments. As above, sites were stratified into hyper- and hypo-methylated CpG sites. For each LOLA analysis, we filtered results retaining enriched region sets only when the support (i.e. number of regions overlapping) >=5 and the *q*-value was <0.05.

To further identify enrichment of CpG sites associated with medically assisted reproduction at cell type-specific histone modifications and DNaseI hypersensitivity sites (DHS) we used eFORGEv2.0 (Breeze *et al.*, 2016). H3 marks and DHS were analysed from the consolidated Roadmap Epigenomics data set. Input was the first 100 CpG sites from the EWAS when ranked by ascending *P*-value. The background set of CpG sites was all CpG sites on the array analyzed in the meta-EWAS. As above, sites were stratified into hyper- and hypo-methylated CpG sites.

The genes mapped to the CpGs associated with medically assisted reproduction at *P*_FDR_<0.05 and in DMRs were searched for published associations in the GWAS catalogue (https://www.ebi.ac.uk/gwas/, v1.0.2: 04 June 2020, date last accessed) (Buniello *et al.*, 2019).

#### Methylation quantitative trait loci analysis

For each CpG associated with medically assisted reproduction in the single-site EWAS at *P*_FDR_<0.05, we searched for methylation quantitative trait loci (mQTLs) in the mQTL database (www.mqtldb.org, 10 June 2020, last date accessed) (Gaunt *et al.*, 2016).

## Results

### Study sample characteristics

The characteristics of the study participants for ALSPAC–ARIES, ALSPAC-MAR and MoBa are shown in Table I and Supplementary Table SII. ALSPAC-MAR and MoBa included a higher proportion of smokers during the first trimester and female offspring compared to ALSPAC-ARIES. Mean birthweight, gestational length and maternal pre-pregnancy BMI were similar across the three study samples. In all three samples, mothers of infants conceived via medically assisted reproduction were older and in ALSPAC-MAR and MoBa infants conceived with medically assisted reproduction were slightly more likely to be female. In ALSPAC-MAR, and to a weaker extent ALSPAC–ARIES, mothers of infants conceived with medically assisted reproduction were less likely to smoke and had lower BMI, which may reflect UK guidelines that restrict some ART procedures to women with a healthy BMI and who do not smoke at the time of treatment.

**Table I:** Characteristics of the three study samples used in the epigenome-wide association studies meta-analysis.

	ALSPAC-ARIES N = 784	ALSPAC-MAR N = 345	MoBa N = 1518
ART (%)	–	–	22 (1.5)
Medically assisted reproduction (%)	28 (3.6)	155 (44.9)	–
Smoked first trimester (%)	100 (12.8)	83 (24.1)	415 (27.3)
Offspring gender at birth (% female)	398 (50.6)	143 (41.4)	706 (46.5)
Maternal age (years) (mean (SD))	29.65 (4.41)	28.84 (4.83)	29.96 (4.31)
Maternal BMI (kg/m^2^) (mean (SD))	22.92 (3.80)	24.52 (6.57)	24.12 (4.32)
Birthweight (g) (mean (SD))	3419 (481)	3364 (606)	3648 (541)
Gestational age (weeks) (mean (SD))	39.70 (1.48)	39.33 (1.86)	39.48 (1.61)

ALSPAC, Avon Longitudinal Study of Parents and Children; ARIES, Accessible Resource for Integrated Epigenomics Studies; MAR, Medically Assisted Reproduction: MoBa, Norwegian Mother, Father, and Child Birth Cohort.

### EWAS meta-analysis

**Figure 1.**
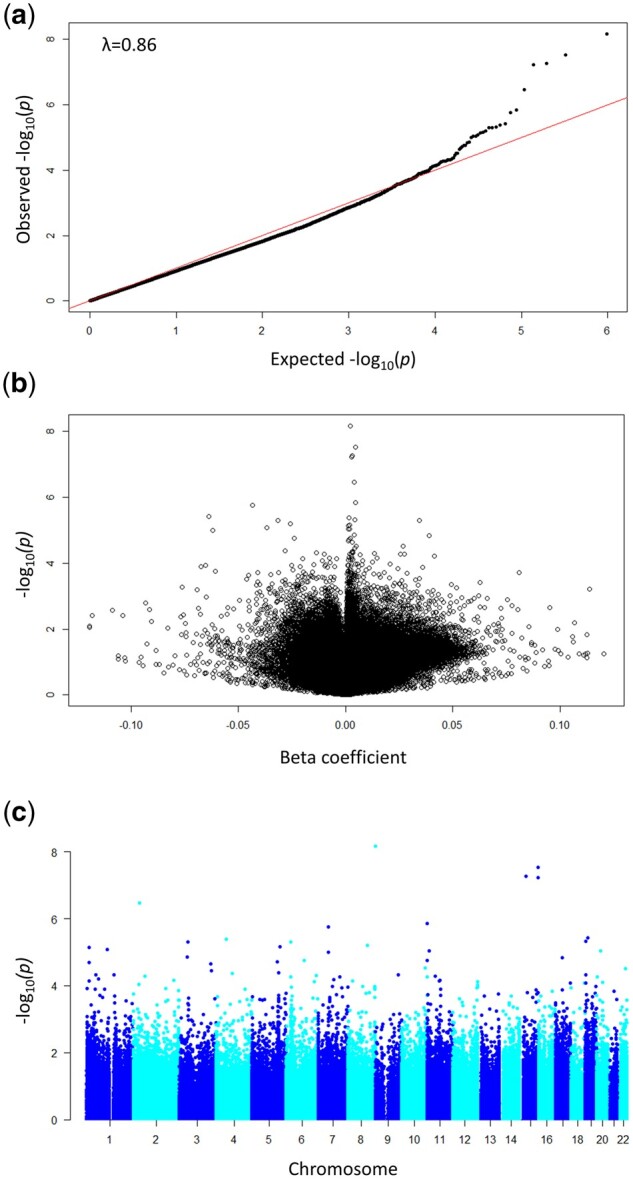
**Association between cord blood DNA methylation and medically assisted reproduction estimated by the meta-analysis.** (**a**) Quantile–quantile plot of the observed versus expected distribution of *P*-values; (**b**) volcano plot of the *P*-values versus the beta coefficients. *λ*= genomic inflation factor calculated using the median method. (**c**) Manhattan plot of the distribution of *P*-values across chromosomes.

Figure 1 and Table II summarize the results of the EWAS meta-analysis adjusted for gender at birth, maternal age at delivery, maternal BMI and maternal smoking during pregnancy. There were five CpG sites associated with medically assisted reproduction at the genome-wide level (*P*_FDR_<0.05), four of which surpassed the threshold for Bonferroni corrected *P*-values (*P*_Bonferroni_<0.05). These were hyper-methylated sites. The effect sizes were lower than 1% absolute difference in methylation between the two groups. The direction of effect was concordant across studies for three of the five sites and it was concordant for four of the five sites between the two ALSPAC subsamples. Three of the CpG sites were located in known genes, including two sites that were annotated to the same gene coding for arrestin domain-containing protein 1 (*ARRDC4*). In all three study samples methylation at *ARRDC4* was <1% higher in ART cases compared to non-ART controls at unadjusted *P*-value < 0.05.

**Table II:** Results of the epigenome-wide association study meta-analysis (CpG sites at *P*_FDR_ < 0.05).

				Meta-analysis					Study-specific results								
									ARIES			MoBa			ALSPAC-MAR		
CpG site	Gene	Chromosome	Position	Beta	SE	*P*-value	*P*_FDR_	*P*_Bonferroni_	Beta	SE	*P*-value	Beta	SE	*P*-value	Beta	SE	*P*-value
cg24051276		8	145561488	0.23	0.04	6.69 × 10^−9^	0.003	0.003	0.46	0.06	1.47 × 10^−12^	0.21	0.12	0.103	0.06	0.05	0.267
cg00012522	*ARRDC4*	15	98503952	0.47	0.08	2.94 × 10^−8^	0.007	0.014	0.73	0.12	2.03 × 10^−09^	0.14	0.13	0.297	0.50	0.25	0.047
cg17855264		15	37180636	0.31	0.06	5.44 × 10^−8^	0.007	0.026	0.74	0.09	3.95 × 10^−15^	0.21	0.14	0.117	−0.04	0.09	0.645
cg17132421	*ARRDC4*	15	98503947	0.30	0.06	5.86 × 10^−8^	0.007	0.028	0.38	0.07	3.28 × 10^−08^	0.07	0.11	0.514	0.31	0.18	0.089
cg18529845	*SRD5A2*	2	31806042	0.41	0.08	3.41 × 10^−07^	0.033	0.164	0.67	0.10	6.25 × 10^−12^	−0.31	0.17	0.069	0.08	0.31	0.807

Beta regression coefficients are reported as percent difference in methylation between groups.

FDR, False discovery Rate; ALSPAC, Avon Longitudinal Study of Parents and Children; ARIES, Accessible Resource for Integrated Epigenomics Studies; MAR, Medically Assisted Reproduction: MoBa, Norwegian Mother, Father, and Child Birth Cohort.

EWAS sensitivity analyses conducted in ALSPAC–ARIES and ALSPAC-MAR with restriction to participants who reported specific forms of medically assisted reproduction (IVF, intrauterine insemination, or OI by hormones) and adjusted for the same covariates as the main analysis showed similar results to the main analyses with high correlation between the regression beta coefficients (*R*^2^= 0.86 for ALSPAC–ARIES and *R*^2^=0.95 for ALSPAC-MAR).

Further sensitivity analyses conducted by MAR procedure within the ALSPAC subsamples (where there were enough cases to run the analysis) showed associations with OI by hormones for all CpGs and intrauterine insemination for cg18529845 (Supplementary Fig. S1).

### Replication in CHART

In the CHART sample, there was no clear evidence of association at any of the 5 CpG sites identified in the discovery meta-analysis (Supplementary Table SIII).

### Biological characterisation

Three of five sites associated with medically assisted reproduction at genome-wide level in our discovery sample were previously reported to be associated with other phenotypes according to the EWAS catalogue (see Supplementary Table SIV). cg18529845 and cg17855264 were associated in multiple EWAS studies of cancer, ageing and HIV infection. cg24051276 was associated with rheumatoid arthritis, dementia and palsy.

No GO terms were shown to be enriched after accounting for multiple testing. In the hypermethylated probe set the most highly enriched terms were biological processes linked to modulation by host of viral process and androgen metabolism (see Supplementary Table SV).

To identify enrichment of sites associated with medically assisted reproduction within gene and gene regulatory regions we utilized eFORGE and LOLA. Using LOLA, enrichment was identified amongst the set of CpG sites that were hypermethylated in cases compared to controls (n = 69 of 100 sites included). Hypermethylated CpGs were enriched for CpG islands (q = 0.0011) (see Supplementary Table SVI). They were also enriched for EZH2 transcription factor binding sites based on ENCODE data. This transcription factor is associated with methylation of H3K27 chromatin leading to chromatin compaction (Laugesen *et al.*, 2019). LOLA analysis also showed that hypermethylated CpG sites were strongly enriched amongst H3K4me2, H3K4me3, H3K9me3 and H3K4me1 in breast and prostate tissues using data derived from the cistrome project.

eFORGE was used to identify cell type specific DHS in tissues profiled by the NIH Roadmap Epigenomics consortium (see Supplementary Table SVII). Enrichment was observed for the hypermethylated set. The strongest enrichment identified for DHS was found in embryonic stem cells. The histone modifications H3K9me3 and H3K9me1 were strongly enriched in multiple tissues, including embryonic stem cells, blood, ovary, stem and foetal cells. H3K9me3 is associated with decreased accessibility of chromatin and is pivotal in lineage commitment during organogenesis and for maintaining cell lineage in differentiated cells (Nicetto *et al.*, 2019). H3K4 methylation was also identified using LOLA and is typically detected in promoter regions of active genes (Barski *et al.*, 2007).

The ‘GWAS Catalogue’ search showed that a genetic variant in *SRD5A2*, whose methylation was associated with medically assisted reproduction, had previously been found to be associated with male baldness (Supplementary Table SVIII).

### mQTL analysis

To explore the potential health consequences of medically assisted reproduction, we searched for *cis*-mQTLs of the top five CpGs from the meta-analysis by looking at SNPs associated with the CpGs in the mQTLdb database (www.mqtldb.org). We were not able to identify any suitable mQTL from this analysis as all the SNPs associated with the five top CpGs were located in *trans* (>1 kb from the CpG site).

## Discussion

We conducted an EWAS meta-analysis of the association of medically assisted reproduction with cord blood DNA methylation across two European birth cohorts, ALSPAC and MoBa. The results showed evidence of an association between medically assisted reproduction and DNA methylation at five CpG sites. By interrogating online databases, we also found evidence that some of the methylation differences associated with medically assisted reproduction were associated with diseases and health-related traits in adulthood. At the molecular level, hypermethylation linked to medically assisted reproduction occurred in regulatory sites involved in chromatin remodelling. However, we could not confirm whether the associations of medically assisted reproduction with differential methylation at the strongest five CpGs were causal (or might be explained by confounding, including by the underlying pathology resulting in infertility). Nor could we establish whether the methylation changes were causally linked to diseases and health-related traits using Mendelian randomization.

Several previous studies have explored differential DNA methylation in infant blood cells related to conception by ART using either candidate or EWAS approaches. Most of these have had smaller sample sizes than our study and/or have not sought replication in an independent group. In a recent study conducted on the CHART cohort used as replication in our study (Novakovic *et al.*, 2019), 18 CpG sites were associated with ART, 9 of which were available in our study. In our meta-analysis, at four of these nine CpGs the direction of the effects was concordant (including at *CHRNE*) and with low *P*-values (0.07–0.006) (Supplementary Table SIX), thus, providing some evidence of consistency across studies, although none of these nine CpGs had *P*-values < 0.006 (*P* = 0.05 Bonferroni-corrected to take into account nine multiple comparisons). Differences between our study and this one may be explained by differences in the adjustment for confounding factors (we adjusted for batch, cell-type, maternal pre-pregnancy BMI, pregnancy smoking, age at delivery and infant sex, whereas the published study adjusted for batch, cell-type and infant sex), the different sample used (blood in our study and Guthrie spots in the published study) and/or differences in the cases definition (broad range of treatments and self-reported in our study, whereas restricted to ART and from hospital records in the published study). The results in the published study are more generally related to ART (which involves OI as part of the procedure and *in vitro* manipulation processes), whereas the associations reported in our study seems to be specific for procedures that did not involve *in vitro* gamete or embryo manipulation (OI and intrauterine insemination). However, given that the two studies were broadly similar in population, analysis methods and size, the lack of consistency between the two may be because the findings in both studies are due to chance and do not reflect real replicable associations. This would be consistent with the lack of replication of our findings in the CHART study.

Most of the CpGs identified in this study are located in the proximity of known genes. We found evidence of hypermethylation at the transcription start site of the *SRD5A2* gene, coding for steroid 5-alpha Reductase 2, an enzyme involved in androgen metabolism and male sexual development. Hypermethylation at this site was associated particularly with OI and intrauterine insemination, suggesting a specific effect of hormonal stimulation, gamete manipulation, and/or freezing and thawing of eggs or sperm. Since our study is observational, we cannot rule out that an impairment in this gene at the parental level was present before having received the medical procedures to help conceiving rather than their consequence and could be related to parental sub-fertility rather than the fertility procedures *per se*. The lack of association in the replication sample at this and the other sites that were differentially methylated in our discovery sample may be due to the different sample collections (cord blood in our discovery meta-analysis and Guthrie dry blood spots collected at 2–4 days in CHART). They could also be due to heterogeneity in the cases definition between cohorts. In CHART, only those conceived by IVF, ICSI or, in a minority of cases, other ART were included in the cases and this was obtained from hospital records. However, the most likely interpretation is that these additional associations are driven by chance.

Our study has several strengths compared to other previously published studies. By combining participants from different studies, we have achieved a larger sample size. We also adjusted for potential confounding maternal characteristics (maternal pre-pregnancy BMI, pregnancy smoking and age at delivery), whereas most previous studies had either smaller sample sizes or adjusted for fewer confounders. Maternal BMI, smoking and age are associated with low fertility as well as DNA methylation and including these factors is important as it partially controls for confounding due to infertility. We also conducted extensive biological characterisation using freely available databases and an mQTL look-up to attempt to determine potential causal links to disease and health-related traits. Finally, we explored replication in an independent sample.

This study has also some limitations. First, our definition of MAR is heterogeneous. OI by hormones, is often used in combination with IVF and intrauterine insemination. Therefore, it is challenging to separate the effects of hormone stimulation from those of *in vitro* culturing. In our discovery meta-analysis our main exposure was self-reported assisted reproduction and, although we could explore in ALSPAC the effects of OI and intrauterine insemination, the IVF groups had very small sample sizes. In ALSPAC, participants were asked a very general question about having used any treatment to help them conceive and then had the opportunity to provide additional information in a text response. In MoBa, the data were obtained from the birth register, but the report was a single entry completed by a midwife that asked whether the conception was by ART (defined as IVF, ICSI or other ART) so we were unable to explore the specific effect of different procedures. Despite our meta-analysis approach, case numbers in each cohort are small and this limitation should be addressed in future studies. Another limitation is that due to the observational nature of the study it has not been possible to establish causality between medically assisted reproduction and offspring DNA methylation. Even though we adjusted for potential confounders the possibility of residual confounding, for example by paternal sub-fertility, remains. Furthermore, due to an insufficient number of mQTLs, our study did not provide the opportunity to conduct Mendelian randomization to explore causal effects between methylation and disease risk.

Overall, we show that newborns conceived by medically assisted reproduction present with altered DNA methylation in cord blood white cells. However, we cannot distinguish whether these methylation profiles are due to the actual fertility treatments, reflect the underlying causes of subfertility, or are chance associations. If any are causal, we do not know whether there are long-term consequences for offspring health. As this is one of the largest studies to date, has adjusted for confounders and tried to explore replication, it highlights the need for larger studies and/or attempts to harmonize data across all studies and undertake a larger collaborative effort. There is also a need for studies with more detailed information on the type of fertility treatment, and on the cause of parental sub-fertility. However, to the best of our knowledge, studies with such detailed exposure information and DNA methylation in large numbers are currently lacking. Additional follow-up studies with DNA methylation at older ages would also be useful to investigate a potential resolution of DNA methylation variation associated with fertility treatments over time as indicated by the CHART study (Novakovic, *et al.*, 2019). Current efforts in generating more mQTL databases such as the GoDMC consortium (http://www.godmc.org.uk/) might provide additional mQTL for use in Mendelian randomisation to establish causal links between differential DNA methylation related to medically assisted reproduction and future disease risks.

## Data availability

The data underlying this article cannot be shared publicly for the privacy of individuals that participated in the study*.* The data will be shared on reasonable request to the individual cohorts. For ALSPAC, data are available according to the procedures listed at http://www.bristol.ac.uk/alspac/researchers/access/. For MoBa, data can be requested at https://www.fhi.no/en/studies/moba/for-forskere-artikler/research-and-data-access/.

## Supplementary Material

deab137_Supplementary_Table_S1

deab137_Supplementary_Table_S2

deab137_Supplementary_Table_S3

deab137_Supplementary_Table_S4

deab137_Supplementary_Table_S5

deab137_Supplementary_Table_S6

deab137_Supplementary_Table_S7

deab137_Supplementary_Table_S8

deab137_Supplementary_Table_S9

deab137_Supplementary_Figure_S1

## References

[deab137-B1] Ashburner M , Ball CA, Blake JA, Botstein D, Butler H, Cherry JM, Davis AP, Dolinski K, Dwight SS, Eppig JT. et al Gene ontology: tool for the unification of biology. The Gene Ontology Consortium. Nat Genet 2000;25:25–29. 10802651 10.1038/75556 PMC3037419

[deab137-B2] Bakulski KM , Feinberg JI, Andrews SV, Yang J, Brown S, L. McKenney S, Witter F, Walston J, Feinberg AP, Fallin MD. DNA methylation of cord blood cell types: applications for mixed cell birth studies. Epigenetics 2016;11:354–362. 27019159 10.1080/15592294.2016.1161875 PMC4889293

[deab137-B3] Barfield RT , Kilaru V, Smith AK, Conneely KN. CpGassoc: an R function for analysis of DNA methylation microarray data. Bioinformatics 2012;28:1280–1281. 22451269 10.1093/bioinformatics/bts124 PMC3577110

[deab137-B4] Barski A , Cuddapah S, Cui K, Roh TY, Schones DE, Wang Z, Wei G, Chepelev I, Zhao K. High-resolution profiling of histone methylations in the human genome. Cell 2007;129:823–837. 17512414 10.1016/j.cell.2007.05.009

[deab137-B5] Bibikova M , Barnes B, Tsan C, Ho V, Klotzle B, Le JM, Delano D, Zhang L, Schroth GP, Gunderson KL. et al High density DNA methylation array with single CpG site resolution. Genomics 2011;98:288–295. 21839163 10.1016/j.ygeno.2011.07.007

[deab137-B6] Boyd A , Golding J, Macleod J, Lawlor DA, Fraser A, Henderson J, Molloy L, Ness A, Ring S, Davey Smith G. Cohort Profile: the ‘children of the 90s’ – the index offspring of the Avon Longitudinal Study of Parents and Children. Int J Epidemiol 2013;42:111–127. 22507743 10.1093/ije/dys064 PMC3600618

[deab137-B7] Breeze CE , Paul DS, van Dongen J, Butcher LM, Ambrose JC, Barrett JE, Lowe R, Rakyan VK, Iotchkova V, Frontini M. et al eFORGE: a tool for identifying cell type-specific signal in epigenomic data. Cell Rep 2016;17:2137–2150. 27851974 10.1016/j.celrep.2016.10.059 PMC5120369

[deab137-B8] Buniello A , MacArthur JAL, Cerezo M, Harris LW, Hayhurst J, Malangone C, McMahon A, Morales J, Mountjoy E, Sollis E. et al The NHGRI-EBI GWAS catalog of published genome-wide association studies, targeted arrays and summary statistics 2019. Nucleic Acids Res 2019;47:D1005–D1012. 30445434 10.1093/nar/gky1120 PMC6323933

[deab137-B9] Canovas S , Ross PJ, Kelsey G, Coy P. DNA methylation in embryo development: epigenetic impact of ART (Assisted Reproductive Technologies). Bioessays 2017;39:1700106. 10.1002/bies.201700106 28940661

[deab137-B10] Cao Z , Zhang M, Xu T, Chen Z, Tong X, Zhang D, Wang Y, Zhang L, Gao D, Luo L. et al Vitrification of murine mature metaphase II oocytes perturbs DNA methylation reprogramming during preimplantation embryo development. Cryobiology 2019;87:91–98. 30707961 10.1016/j.cryobiol.2019.01.012

[deab137-B11] Castillo-Fernandez JE , Loke YJ, Bass-Stringer S, Gao F, Xia Y, Wu H, Lu H, Liu Y, Wang J, Spector TD. et al DNA methylation changes at infertility genes in newborn twins conceived by in vitro fertilisation. Genome Med 2017;9:28. 28340599 10.1186/s13073-017-0413-5 PMC5364659

[deab137-B12] Chen M , Heilbronn LK. The health outcomes of human offspring conceived by assisted reproductive technologies (ART). J Dev Orig Health Dis 2017;8:388–402. 28416029 10.1017/S2040174417000228

[deab137-B13] Choufani S , Turinsky AL, Melamed N, Greenblatt E, Brudno M, Berard A, Fraser WD, Weksberg R, Trasler J, Monnier P; 3D Cohort Study Group. Impact of assisted reproduction, infertility, sex and paternal factors on the placental DNA methylome. Hum Mol Genet 2019;28:372–385. 30239726 10.1093/hmg/ddy321 PMC6337702

[deab137-B14] Davies MJ , Moore VM, Willson KJ, Van Essen P, Priest K, Scott H, Haan EA, Chan A. Reproductive technologies and the risk of birth defects. N Engl J Med 2012;366:1803–1813. 22559061 10.1056/NEJMoa1008095

[deab137-B15] Doherty AS , Mann MR, Tremblay KD, Bartolomei MS, Schultz RM. Differential effects of culture on imprinted H19 expression in the preimplantation mouse embryo. Biol Reprod 2000;62:1526–1535. 10819752 10.1095/biolreprod62.6.1526

[deab137-B16] El Hajj N , Haertle L, Dittrich M, Denk S, Lehnen H, Hahn T, Schorsch M, Haaf T. DNA methylation signatures in cord blood of ICSI children. Hum Reprod 2017;32:1761–1769. 28575352 10.1093/humrep/dex209 PMC5850272

[deab137-B17] Elias FTS , Weber-Adrian D, Pudwell J, Carter J, Walker M, Gaudet L, Smith G, Velez MP. Neonatal outcomes in singleton pregnancies conceived by fresh or frozen embryo transfer compared to spontaneous conceptions: a systematic review and meta-analysis. Arch Gynecol Obstet 2020;302:31–45. 32445067 10.1007/s00404-020-05593-4 PMC7266861

[deab137-B18] Estill MS , Bolnick JM, Waterland RA, Bolnick AD, Diamond MP, Krawetz SA. Assisted reproductive technology alters deoxyribonucleic acid methylation profiles in bloodspots of newborn infants. Fertil Steril 2016;106:629–639 e610. 27288894 10.1016/j.fertnstert.2016.05.006

[deab137-B19] Fraser A , Macdonald-Wallis C, Tilling K, Boyd A, Golding J, Davey Smith G, Henderson J, Macleod J, Molloy L, Ness A. et al Cohort profile: the Avon Longitudinal Study of parents and children: ALSPAC mothers cohort. Int J Epidemiol 2013;42:97–110. 22507742 10.1093/ije/dys066 PMC3600619

[deab137-B20] Gaunt TR , Shihab HA, Hemani G, Min JL, Woodward G, Lyttleton O, Zheng J, Duggirala A, McArdle WL, Ho K. et al Systematic identification of genetic influences on methylation across the human life course. Genome Biol 2016;17:61. 27036880 10.1186/s13059-016-0926-z PMC4818469

[deab137-B21] Geeleher P , Hartnett L, Egan LJ, Golden A, Raja Ali RA, Seoighe C. Gene-set analysis is severely biased when applied to genome-wide methylation data. Bioinformatics 2013;29:1851–1857. 23732277 10.1093/bioinformatics/btt311

[deab137-B22] Gentilini D , Somigliana E, Pagliardini L, Rabellotti E, Garagnani P, Bernardinelli L, Papaleo E, Candiani M, Di Blasio AM, Viganò P. Multifactorial analysis of the stochastic epigenetic variability in cord blood confirmed an impact of common behavioral and environmental factors but not of in vitro conception. Clin Epigenet 2018;10:77. 10.1186/s13148-018-0510-3 PMC5994106 29930742

[deab137-B23] Haberg SE , London SJ, Nafstad P, Nilsen RM, Ueland PM, Vollset SE, Nystad W. Maternal folate levels in pregnancy and asthma in children at age 3 years. J Allergy Clin Immunol 2011;127:262–264, 264 e261. 21094522 10.1016/j.jaci.2010.10.004 PMC3108064

[deab137-B24] Halliday J , Lewis S, Kennedy J, Burgner DP, Juonala M, Hammarberg K, Amor DJ, Doyle LW, Saffery R, Ranganathan S. et al Health of adults aged 22 to 35 years conceived by assisted reproductive technology. Fertil Steril 2019;112:130–139. 31003618 10.1016/j.fertnstert.2019.03.001

[deab137-B25] Hansen M , Kurinczuk JJ, Milne E, de Klerk N, Bower C. Assisted reproductive technology and birth defects: a systematic review and meta-analysis. Hum Reprod Update 2013;19:330–353. 23449641 10.1093/humupd/dmt006

[deab137-B26] Joubert BR , Haberg SE, Nilsen RM, Wang X, Vollset SE, Murphy SK, Huang Z, Hoyo C, Midttun O, Cupul-Uicab LA. et al 450K epigenome-wide scan identifies differential DNA methylation in newborns related to maternal smoking during pregnancy. Environ Health Perspect 2012;120:1425–1431. 22851337 10.1289/ehp.1205412 PMC3491949

[deab137-B27] Kallen B , Finnstrom O, Lindam A, Nilsson E, Nygren KG, Otterblad PO. Congenital malformations in infants born after in vitro fertilization in Sweden. Birth Defects Res A Clin Mol Teratol 2010;88:137–143. 20063307 10.1002/bdra.20645

[deab137-B28] Katari S , Turan N, Bibikova M, Erinle O, Chalian R, Foster M, Gaughan JP, Coutifaris C, Sapienza C. DNA methylation and gene expression differences in children conceived in vitro or in vivo. Hum Mol Genet 2009;18:3769–3778. 19605411 10.1093/hmg/ddp319 PMC2748887

[deab137-B29] Laugesen A , Hojfeldt JW, Helin K. Molecular mechanisms directing PRC2 recruitment and H3K27 methylation. Mol Cell 2019;74:8–18. 30951652 10.1016/j.molcel.2019.03.011 PMC6452890

[deab137-B30] Lawlor DA , Nelson SM. Effect of age on decisions about the numbers of embryos to transfer in assisted conception: a prospective study. Lancet 2012;379:521–527. 22243709 10.1016/S0140-6736(11)61267-1

[deab137-B31] Lazaraviciute G , Kauser M, Bhattacharya S, Haggarty P, Bhattacharya S. A systematic review and meta-analysis of DNA methylation levels and imprinting disorders in children conceived by IVF/ICSI compared with children conceived spontaneously. Hum Reprod Update 2014;20:840–852. 24961233 10.1093/humupd/dmu033

[deab137-B32] Levi Setti PE , Moioli M, Smeraldi A, Cesaratto E, Menduni F, Livio S, Morenghi E, Patrizio P. Obstetric outcome and incidence of congenital anomalies in 2351 IVF/ICSI babies. J Assist Reprod Genet 2016;33:711–717. 27116010 10.1007/s10815-016-0714-4 PMC4889486

[deab137-B33] Lewis S , Kennedy J, Burgner D, McLachlan R, Ranganathan S, Hammarberg K, Saffery R, Amor DJ, Cheung MMH, Doyle LW. et al Clinical review of 24-35 year olds conceived with and without in vitro fertilization: study protocol. Reprod Health 2017;14:117. 28931409 10.1186/s12978-017-0377-3 PMC5607609

[deab137-B34] Magnus P , Birke C, Vejrup K, Haugan A, Alsaker E, Daltveit AK, Handal M, Haugen M, Hoiseth G, Knudsen GP. et al Cohort profile update: The Norwegian Mother and Child Cohort Study (MoBa). Int J Epidemiol 2016;45:382–388. 27063603 10.1093/ije/dyw029

[deab137-B35] Magnus P , Irgens LM, Haug K, Nystad W, Skjaerven R, Stoltenberg C, MoBa SG, MoBa Study Group. Cohort profile: the Norwegian Mother and Child Cohort Study (MoBa). Int J Epidemiol 2006;35:1146–1150. 16926217 10.1093/ije/dyl170

[deab137-B36] Maksimovic J , Gordon L, Oshlack A. SWAN: subset-quantile within array normalization for illumina infinium HumanMethylation450 BeadChips. Genome Biol 2012;13:R44. 22703947 10.1186/gb-2012-13-6-r44 PMC3446316

[deab137-B37] Mann MR , Lee SS, Doherty AS, Verona RI, Nolen LD, Schultz RM, Bartolomei MS. Selective loss of imprinting in the placenta following preimplantation development in culture. Development 2004;131:3727–3735. 15240554 10.1242/dev.01241

[deab137-B38] Market-Velker BA , Fernandes AD, Mann MR. Side-by-side comparison of five commercial media systems in a mouse model: suboptimal in vitro culture interferes with imprint maintenance. Biol Reprod2010a;83:938–950. 20702853 10.1095/biolreprod.110.085480

[deab137-B39] Market-Velker BA , Zhang L, Magri LS, Bonvissuto AC, Mann MR. Dual effects of superovulation: loss of maternal and paternal imprinted methylation in a dose-dependent manner. Hum Mol Genet2010b;19:36–51. 19805400 10.1093/hmg/ddp465

[deab137-B40] *Market-Velker BA , Denomme MM, Mann MR. Loss of genomic imprinting in mouse embryos with fast rates of preimplantation development in culture. Biol Reprod 2012;86:141–116. 22278980 10.1095/biolreprod.111.096602 PMC4480067

[deab137-B41] Melamed N , Choufani S, Wilkins-Haug LE, Koren G, Weksberg R. Comparison of genome-wide and gene-specific DNA methylation between ART and naturally conceived pregnancies. Epigenetics 2015;10:474–483. 25580569 10.4161/15592294.2014.988041 PMC4623493

[deab137-B42] Min JL , Hemani G, Davey Smith G, Relton C, Suderman M. Meffil: efficient normalization and analysis of very large DNA methylation datasets. Bioinformatics 2018;34:3983–3989. 29931280 10.1093/bioinformatics/bty476 PMC6247925

[deab137-B43] Nelson SM , Lawlor DA. Predicting live birth, preterm delivery, and low birth weight in infants born from in vitro fertilisation: a prospective study of 144,018 treatment cycles. PLoS Med 2011;8:e1000386. 21245905 10.1371/journal.pmed.1000386 PMC3014925

[deab137-B44] Nicetto D , Donahue G, Jain T, Peng T, Sidoli S, Sheng L, Montavon T, Becker JS, Grindheim JM, Blahnik K. et al H3K9me3-heterochromatin loss at protein-coding genes enables developmental lineage specification. Science 2019;363:294–297. 30606806 10.1126/science.aau0583 PMC6664818

[deab137-B45] Novakovic B , Lewis S, Halliday J, Kennedy J, Burgner DP, Czajko A, Kim B, Sexton-Oates A, Juonala M, Hammarberg K. et al Assisted reproductive technologies are associated with limited epigenetic variation at birth that largely resolves by adulthood. Nat Commun 2019;10:3922. 31477727 10.1038/s41467-019-11929-9 PMC6718382

[deab137-B46] Paltiel L , Anita H, Skjerden T, Harbak K, Bækken S, Nina Kristin S, Knudsen GP, Magnus P. The biobank of the Norwegian Mother and Child Cohort Study – present status. Nor J Epidemiol 2014;24:29–35.

[deab137-B47] Phipson B , Maksimovic J, Oshlack A. missMethyl: an R package for analyzing data from Illumina's HumanMethylation450 platform. Bioinformatics 2016;32:286–288. 26424855 10.1093/bioinformatics/btv560

[deab137-B48] Reese SE , Xu CJ, den Dekker HT, Lee MK, Sikdar S, Ruiz-Arenas C, Merid SK, Rezwan FI, Page CM, Ullemar V. et al Epigenome-wide meta-analysis of DNA methylation and childhood asthma. J Allergy Clin Immunol 2019;143:2062–2074. 30579849 10.1016/j.jaci.2018.11.043 PMC6556405

[deab137-B49] Relton CL , Gaunt T, McArdle W, Ho K, Duggirala A, Shihab H, Woodward G, Lyttleton O, Evans DM, Reik W. et al Data resource profile: Accessible Resource for Integrated Epigenomic Studies (ARIES). Int J Epidemiol 2015;44:1181–1190. 25991711 10.1093/ije/dyv072 PMC5593097

[deab137-B50] Rice K , Higgins JPT, Lumley T. A re-evaluation of fixed effect(s) meta-analysis. J R Stat Soc A 2018;181:205–227.

[deab137-B51] Sakian S , Louie K, Wong EC, Havelock J, Kashyap S, Rowe T, Taylor B, Ma S. Altered gene expression of H19 and IGF2 in placentas from ART pregnancies. Placenta 2015;36:1100–1105. 26386650 10.1016/j.placenta.2015.08.008

[deab137-B52] Sheffield NC , Bock C. LOLA: enrichment analysis for genomic region sets and regulatory elements in R and Bioconductor. Bioinformatics 2016;32:587–589. 26508757 10.1093/bioinformatics/btv612 PMC4743627

[deab137-B53] Song S , Ghosh J, Mainigi M, Turan N, Weinerman R, Truongcao M, Coutifaris C, Sapienza C. DNA methylation differences between in vitro- and in vivo-conceived children are associated with ART procedures rather than infertility. Clin Epigenet 2015;7:41. 10.1186/s13148-015-0071-7 PMC4404660 25901188

[deab137-B54] The Gene Ontology Consortium. Expansion of the Gene Ontology knowledgebase and resources. Nucleic Acids Res 2017;45:D331–D338. 27899567 10.1093/nar/gkw1108 PMC5210579

[deab137-B55] Tierling S , Souren NY, Gries J, Loporto C, Groth M, Lutsik P, Neitzel H, Utz-Billing I, Gillessen-Kaesbach G, Kentenich H. et al Assisted reproductive technologies do not enhance the variability of DNA methylation imprints in human. J Med Genet 2010;47:371–376. 19948534 10.1136/jmg.2009.073189

[deab137-B56] Van Heertum K , Weinerman R. Neonatal outcomes following fresh as compared to frozen/thawed embryo transfer in in vitro fertilization. Birth Defects Res 2018;110:625–629. 29493111 10.1002/bdr2.1216

[deab137-B57] Velker BAM , Denomme MM, Krafty RT, Mann MRW. High frequency of imprinted methylation errors in human preimplantation. Environ Epigenet 2017;5:17311. 10.1038/srep17311 PMC4667293 26626153

[deab137-B58] White CR , Denomme MM, Tekpetey FR, Feyles V, Power SG, Mann MR. High frequency of imprinted methylation errors in human preimplantation embryos. Sci Rep 2015;5:17311. 26626153 10.1038/srep17311 PMC4667293

[deab137-B59] Willer CJ , Li Y, Abecasis GR. METAL: fast and efficient meta-analysis of genomewide association scans. Bioinformatics 2010;26:2190–2191. 20616382 10.1093/bioinformatics/btq340 PMC2922887

[deab137-B60] Zegers-Hochschild F , Adamson GD, Dyer S, Racowsky C, de Mouzon J, Sokol R, Rienzi L, Sunde A, Schmidt L, Cooke ID. et al The international glossary on infertility and fertility care, 2017. Hum Reprod 2017;32:1786–1801. 29117321 10.1093/humrep/dex234 PMC5850297

