# Supplementary material for: Association of medically assisted reproduction with offspring cord blood DNA methylation across cohorts
Source: Hum Reprod. 2021 Jun 17;36(8):2403–13. doi: 10.1093/humrep/deab137 (PMC8289315; doi:10.1093/humrep/deab137)
Supplement: deab137_Supplementary_Table_S1 [file deab137_supplementary_table_s1.pdf]

Human Reproduction, pp. 1–1, 2021
doi:10.1093/humrep/deab137

human reproduction

SUPPLEMENTARY DATA

Supplementary Table SI Distribution of other phenotypes in the ALSPAC-MAR sample.

		Natural conception N = 190	Medically assisted reproduction N = 155
**Maternal phenotype**			
Arthritis	No	167	149
	Yes	16	<5
Diabetes history	No	175	154
	Yes	15	<5
Eating disorder	No	179	143
	Yes	22	6
Preeclampsia	No	146	149
	Yes	42	5
Hypertension history	No	145	142
	Yes	41	13
Maternal BMI	Mean (SD)	25.88 (7.52)	22.85 (4.67)
**Offspring phenotype**			
Autism	No	87	98
	Yes	24	<5
Undescended testicle	No	47	34
	Yes	<5	0
