# Supplementary material for: Association of medically assisted reproduction with offspring cord blood DNA methylation across cohorts
Source: Hum Reprod. 2021 Jun 17;36(8):2403–13. doi: 10.1093/humrep/deab137 (PMC8289315; doi:10.1093/humrep/deab137)
Supplement: deab137_Supplementary_Table_S2 [file deab137_supplementary_table_s2.pdf]

Human Reproduction, pp. 1–1, 2021
doi:10.1093/humrep/deab137

human reproduction

SUPPLEMENTARY DATA

Supplementary Table SII **Sample characteristics of samples included in the meta-epigenome-wide association study.**

		ALSPAC-ARIES			ALSPAC-MAR			MoBa		
		Natural	MAR	*P*-value*	Natural	MAR	*P*-value*	Natural	MAR	*P*-value**
**Offspring gender at birth**	Male	369	18	0.16	124	78	0.0071	806	6	0.013
	Female	387	10		66	77		690	16	
**Smoked during pregnancy**	No	658	<5	0.53	130	132	0.0005	1086	17	0.62
	Yes	98	<5		60	23		410	5	
**BMI**	Mean (SD)	22.93 (3.85)	22.58 (2.03)	0.39	25.88 (7.52)	22.85 (4.67)	6.54E−06	24.13 (4.33)	23.72 (3.55)	0.66
**Birthweight (g)**	Mean (SD)	3496 (644)	3358 (599)	0.24	3406 (634)	3312 (552)	0.15	3649 (544)	3534 (293)	0.32
**Gestation (weeks)**	Mean (SD)	39.63 (1.46)	39.14 (1.48)	0.1	39.34 (1.94)	39.32 (1.77)	0.94	39.48 (1.62)	39.54 (1.14)	0.86
**Maternal age delivery (years)**	Mean (SD)	29.59 (4.41)	31.21 (4.08)	0.05	27.90 (5.23)	30.00 (4.01)	3.10E−05	29.92 (4.26)	32.27 (4.61)	0.01
**Sample type**	Blood spots	136	9	0.1	29	73	2.51E−10			
	White cells	620	19		161	82				

**P*-values for categorical variables calculated with https://www.socscistatistics.com/tests/chisquare/Default2.aspx.
***P*-values for continuous variables calculated with https://www.medcalc.org/calc/comparison_of_means.php.
ALSPAC, Avon Longitudinal Study of Parents and Children; ARIES, Accessible Resource for Integrated Epigenomics Studies; MAR, Medically Assisted Reproduction: MoBa, Norwegian Mother, Father, and Child Birth Cohort.
