# Supplementary material for: Association of medically assisted reproduction with offspring cord blood DNA methylation across cohorts
Source: Hum Reprod. 2021 Jun 17;36(8):2403–13. doi: 10.1093/humrep/deab137 (PMC8289315; doi:10.1093/humrep/deab137)
Supplement: deab137_Supplementary_Table_S3 [file deab137_supplementary_table_s3.pdf]

Human Reproduction, pp. 1–1, 2021
doi:10.1093/humrep/deab137

human reproduction

SUPPLEMENTARY DATA

Supplementary Table SIII **Meta-epigenome-wide association study replication in the CHART cohort.**

CpG	Gene	No-ART mean	ART mean	No-ART SE	ART SE	Mean difference	*P*-value
cg00012522	*ARRDC4*	7.1	6.9	0.2	0.1	−0.2	0.16
cg17132421	*ARRDC4*	5.4	5.3	0.1	0.1	0.0	0.39
cg17855264		5.0	4.7	0.2	0.1	−0.3	0.09
cg18529845	*SRD5A2*	8.3	7.6	0.5	0.2	−0.7	0.17
cg24051276		4.5	4.5	0.1	0.1	0.0	0.83
