# Supplementary material for: Association of medically assisted reproduction with offspring cord blood DNA methylation across cohorts
Source: Hum Reprod. 2021 Jun 17;36(8):2403–13. doi: 10.1093/humrep/deab137 (PMC8289315; doi:10.1093/humrep/deab137)
Supplement: deab137_Supplementary_Table_S4 [file deab137_supplementary_table_s4.pdf]

Human Reproduction, pp. 1–1, 2021
doi:10.1093/humrep/deab137

human reproduction

SUPPLEMENTARY DATA

Supplementary Table SIV **CpG sites reported in the meta-epigenome-wide association study catalogue (nr = not reported).**

Author	PMID	Date	Trait	Exposure	N	CpG	Location	Chr	Pos	Gene	Beta	SE	*P*
Liu Y	23334450	1/20/2013	Rheumatoid arthritis	Rheumatoid arthritis	691	cg24051276	chr8:145561488	8	145561488		0.005	0.0004	9.72E−08
Wozniak MB	23526956	3/5/2013	Clear cell renal carcinoma	Clear cell renal carcinoma	129	cg18529845	chr2:31806042	2	31806042	*SRD5A2*	nr	nr	3.88E−16
Wozniak MB	23526956	3/5/2013	Clear cell renal carcinoma	Clear cell renal carcinoma	129	cg17855264	chr15:37180636	15	37180636		nr	nr	1.16E−17
Nones K	24500968	5/9/2014	Pancreatic ductal adenocarcinoma	Pancreatic ductal adenocarcinoma	196	cg18529845	chr2:31806042	2	31806042	*SRD5A2*	nr	nr	2.91E−21
Li Y	24603599	3/6/2014	Frontotemporal dementia	Frontotemporal dementia	148	cg24051276	chr8:145561488	8	145561488		nr	nr	0.374
Li Y	24603599	3/6/2014	Frontotemporal dementia	Frontotemporal dementia	165	cg24051276	chr8:145561488	8	145561488		nr	nr	0.723
Li Y	24603599	3/6/2014	Progressive supranu-clear palsy	Progressive supranu-clear palsy	228	cg24051276	chr8:145561488	8	145561488		nr	nr	0.0139
Marttila S	25888029	3/14/2015	Ageing	Age group	143	cg18529845	chr2:31806042	2	31806042	*SRD5A2*	0.724	0.1031	2.26E−12
Gross AM	27105112	4/21/2016	HIV infection	HIV infection	181	cg18529845	chr2:31806042	2	31806042	*SRD5A2*	1.285	0.2794	4.20E−06
Gross AM	27105112	4/21/2016	HIV infection	HIV infection	181	cg17855264	chr15:37180636	15	37180636		0.803	0.1796	7.91E−06
Xu CJ	28056824	1/5/2017	Age 4 vs age 0	Age 4 vs age 0	269	cg17855264	chr15:37180636	15	37180636		nr	nr	2.48E−14
