# Supplementary material for: Association of medically assisted reproduction with offspring cord blood DNA methylation across cohorts
Source: Hum Reprod. 2021 Jun 17;36(8):2403–13. doi: 10.1093/humrep/deab137 (PMC8289315; doi:10.1093/humrep/deab137)
Supplement: deab137_Supplementary_Table_S5 [file deab137_supplementary_table_s5.pdf]

Human Reproduction, pp. 1–2, 2021
doi:10.1093/humrep/deab137

human reproduction

SUPPLEMENTARY DATA

Supplementary Table SV GO term enrichments for hyper- and hypo- methylated CpG sites identified in meta-epigenome-wide association study.

GO term enrichment using hyper-methylated CpGs amongst the first 100 CpG sites ranked by ascending *P*-value (n = 69)

	Term	Ont	N	DE	P.DE	FDR
GO:0044794	BP	Positive regulation by host of viral process	16	3	1.95E−05	0.445101
GO:0044788	BP	Modulation by host of viral process	29	3	8.29E−05	0.948715
GO:2000809	BP	Positive regulation of synaptic vesicle clustering	5	2	0.000166	1
GO:0006702	BP	Androgen biosynthetic process	11	2	0.000284	1
GO:0051817	BP	Modification of morphology or physiology of other organism involved in symbiotic interaction	112	4	0.000379	1
GO:2000807	BP	Regulation of synaptic vesicle clustering	9	2	0.000635	1
GO:0035821	BP	modification of morphology or physiology of other organism	160	4	0.000885	1
GO:0097104	BP	Postsynaptic membrane assembly	12	2	0.001053	1
GO:0051851	BP	Modification by host of symbiont morphology or physiology	77	3	0.001386	1
GO:0051592	BP	Response to calcium ion	146	4	0.001556	1
GO:0051702	BP	Interaction with symbiont	82	3	0.001667	1
GO:0097091	BP	Synaptic vesicle clustering	16	2	0.00195	1
GO:1901585	BP	Regulation of acid-sensing ion channel activity	1	1	0.002382	1
GO:0008209	BP	Androgen metabolic process	28	2	0.002537	1
GO:0072341	MF	Modified amino acid binding	86	3	0.002808	1
GO:0071277	BP	Cellular response to calcium ion	82	3	0.002948	1
GO:0009917	MF	Sterol 5-alpha reductase activity	1	1	0.002972	1
GO:0035597	MF	N6-isopentenyladenosine methylthiotransferase activity	1	1	0.003066	1
GO:0048792	BP	Spontaneous exocytosis of neurotransmitter	1	1	0.003272	1
GO:0002174	BP	Mammary stem cell proliferation	1	1	0.003307	1

GO term enrichment using hypo-methylated CpGs amongst the first 100 CpG sites ranked by ascending *P* value (n = 31)

	Term	Ont	N	DE	P.DE	FDR
GO:0042396	BP	Phosphagen biosynthetic process	5	1	0.002	1
GO:0006599	BP	Phosphagen metabolic process	5	1	0.002	1
GO:0046314	BP	Phosphocreatine biosynthetic process	5	1	0.002	1
GO:0006603	BP	Phosphocreatine metabolic process	5	1	0.002	1
GO:0006649	BP	Phospholipid transfer to membrane	2	1	0.0027	1
GO:0052026	BP	Modulation by symbiont of host transcription	1	1	0.003	1
GO:0019056	BP	Modulation by virus of host transcription	1	1	0.003	1
GO:0039656	BP	Modulation by virus of host gene expression	3	1	0.0033	1
GO:0003952	MF	NAD+ synthase (glutamine-hydrolyzing) activity	1	1	0.0035	1
GO:0008622	CC	Epsilon DNA polymerase complex	5	1	0.0039	1
GO:0090555	MF	Phosphatidylethanolamine-translocating ATPase activity	2	1	0.0041	1
GO:0097733	CC	Photoreceptor cell cilium	112	2	0.0043	1
GO:0097731	CC	9 + 0 non-motile cilium	114	2	0.0044	1
GO:0004359	MF	Glutaminase activity	3	1	0.0044	1
GO:0045004	BP	DNA replication proofreading	2	1	0.0047	1
GO:0008310	MF	Single-stranded DNA 3'–5' exodeoxyribonuclease activity	6	1	0.0048	1
GO:0006600	BP	Creatine metabolic process	8	1	0.0049	1
GO:0006272	BP	Leading strand elongation	3	1	0.0049	1

(continued)

Human Reproduction, pp. 1–2, 2021
doi:10.1093/humrep/deab137

human reproduction

SUPPLEMENTARY DATA

Supplementary Table SV Continued

GO term enrichment using hypo-methylated CpGs amongst the first 100 CpG sites ranked by ascending *P* value (n = 31)						
	**Term**	**Ont**	**N**	**DE**	**P.DE**	**FDR**
GO:1904121	MF	Phosphatidylethanolamine transporter activity	3	1	0.0052	1
GO:0004111	MF	Creatine kinase activity	6	1	0.0054	1
**GO term enrichment using the first 100 CpG sites ranked by ascending P value**						
	**Term**	**Ont**	**N**	**DE**	**P.DE**	**FDR**
GO:0044794	BP	Positive regulation by host of viral process	16	3	4.69E−05	1
GO:0051817	BP	Modification of morphology or physiology of other organism involved in symbiotic interaction	112	5	9.11E−05	1
GO:0044788	BP	Modulation by host of viral process	29	3	0.00019	1
GO:0035821	BP	Modification of morphology or physiology of other organism	160	5	0.0002361	1
GO:2000809	BP	Positive regulation of synaptic vesicle clustering	5	2	0.0003716	1
GO:0006702	BP	Androgen biosynthetic process	11	2	0.0005193	1
GO:0099068	BP	Postsynapse assembly	30	3	0.0008634	1
GO:2000807	BP	Regulation of synaptic vesicle clustering	9	2	0.0014622	1
GO:0097104	BP	Postsynaptic membrane assembly	12	2	0.0024321	1
GO:1901585	BP	Regulation of acid-sensing ion channel activity	1	1	0.002454	1
GO:0051851	BP	Modification by host of symbiont morphology or physiology	77	3	0.0029057	1
GO:0006271	BP	DNA strand elongation involved in DNA replication	20	2	0.0030088	1
GO:0097381	CC	Photoreceptor disc membrane	23	2	0.0031191	1
GO:0051702	BP	Interaction with symbiont	82	3	0.0034682	1
GO:0035597	MF	N6-isopentenyladenosine methylthiotransferase activity	1	1	0.0036888	1
GO:0009917	MF	Sterol 5-alpha reductase activity	1	1	0.0037508	1
GO:0048792	BP	Spontaneous exocytosis of neurotransmitter	1	1	0.0038042	1
GO:0006297	BP	Nucleotide-excision repair, DNA gap filling	23	2	0.0041488	1
GO:0097091	BP	Synaptic vesicle clustering	16	2	0.0043277	1
GO:0002174	BP	Mammary stem cell proliferation	1	1	0.0045403	1
