# Supplementary material for: Association of medically assisted reproduction with offspring cord blood DNA methylation across cohorts
Source: Hum Reprod. 2021 Jun 17;36(8):2403–13. doi: 10.1093/humrep/deab137 (PMC8289315; doi:10.1093/humrep/deab137)
Supplement: deab137_Supplementary_Table_S8 [file deab137_supplementary_table_s8.pdf]

Human Reproduction, pp. 1–1, 2021
doi:10.1093/humrep/deab137

human reproduction

SUPPLEMENTARY DATA

Supplementary Table SVIII Associations found in the GWAS catalog pertaining genes mapped to the top CpG sites of the meta-analysis.

Pubmed ID	First Author	Date	Disease Trait	Chromosome	Position	Mapped Gene	SNP	*P*-value
28196072	Hagenaars SP	2/14/2017	Male-pattern baldness	2	31580756	*SRD5A2*	rs9282858	2.00E−23
27182965	Pickrell JK	5/16/2016	Male-pattern baldness	2	31580756	*SRD5A2*	rs9282858	2.00E−15
28272467	Heilmann-Heimbach S	3/8/2017	Male−pattern baldness	2	31580756	*SRD5A2*	rs9282858	9.00E-18
