# Supplementary material for: Association of medically assisted reproduction with offspring cord blood DNA methylation across cohorts
Source: Hum Reprod. 2021 Jun 17;36(8):2403–13. doi: 10.1093/humrep/deab137 (PMC8289315; doi:10.1093/humrep/deab137)
Supplement: deab137_Supplementary_Table_S9 [file deab137_supplementary_table_s9.pdf]

Human Reproduction, pp. 1–1, 2021
doi:10.1093/humrep/deab137

human reproduction

SUPPLEMENTARY DATA

Supplementary Table SIX **Look-up of top 18 CpG sites from Novakovic *et al.* (2019) as reported in Supplementary Data S1, in the current meta-analysis.**

CpG	Direction CHART	Effect* meta-analysis	SD meta-analysis	*P*-value meta-analysis	Direction by study in the meta-analysis	UCSC gene name
cg10553748	−	−0.0165	0.006	0.006	+—	*CHRNE*
cg03904042	−	−0.033	0.0123	0.007	—−	*NECAB3*
cg24768135	−	−0.0231	0.0113	0.041	+—	*CHRNE*
cg16761754	−	−0.0198	0.0111	0.073	—−	
cg12610079	+	−0.0057	0.0035	0.103	—−	*C1orf110*
cg02406531	−	0.0048	0.0063	0.439	−++	
cg04471375	+	−0.0031	0.0043	0.467	+—	*GOT2*
cg26536949	+	−0.0083	0.0255	0.746	+—	
cg10318313	+	−0.0004	0.0042	0.921	+-+	*NAPIL4*

*Difference in proportion methylation.
