# Supplementary material for: Association of medically assisted reproduction with offspring cord blood DNA methylation across cohorts
Source: Hum Reprod. 2021 Jun 17;36(8):2403–13. doi: 10.1093/humrep/deab137 (PMC8289315; doi:10.1093/humrep/deab137)
Supplement: deab137_Supplementary_Figure_S1 [file deab137_supplementary_figure_s1.pdf]

Human Reproduction, pp. 1–1, 2021
doi:10.1093/humrep/deab137

human reproduction **SUPPLEMENTARY DATA**

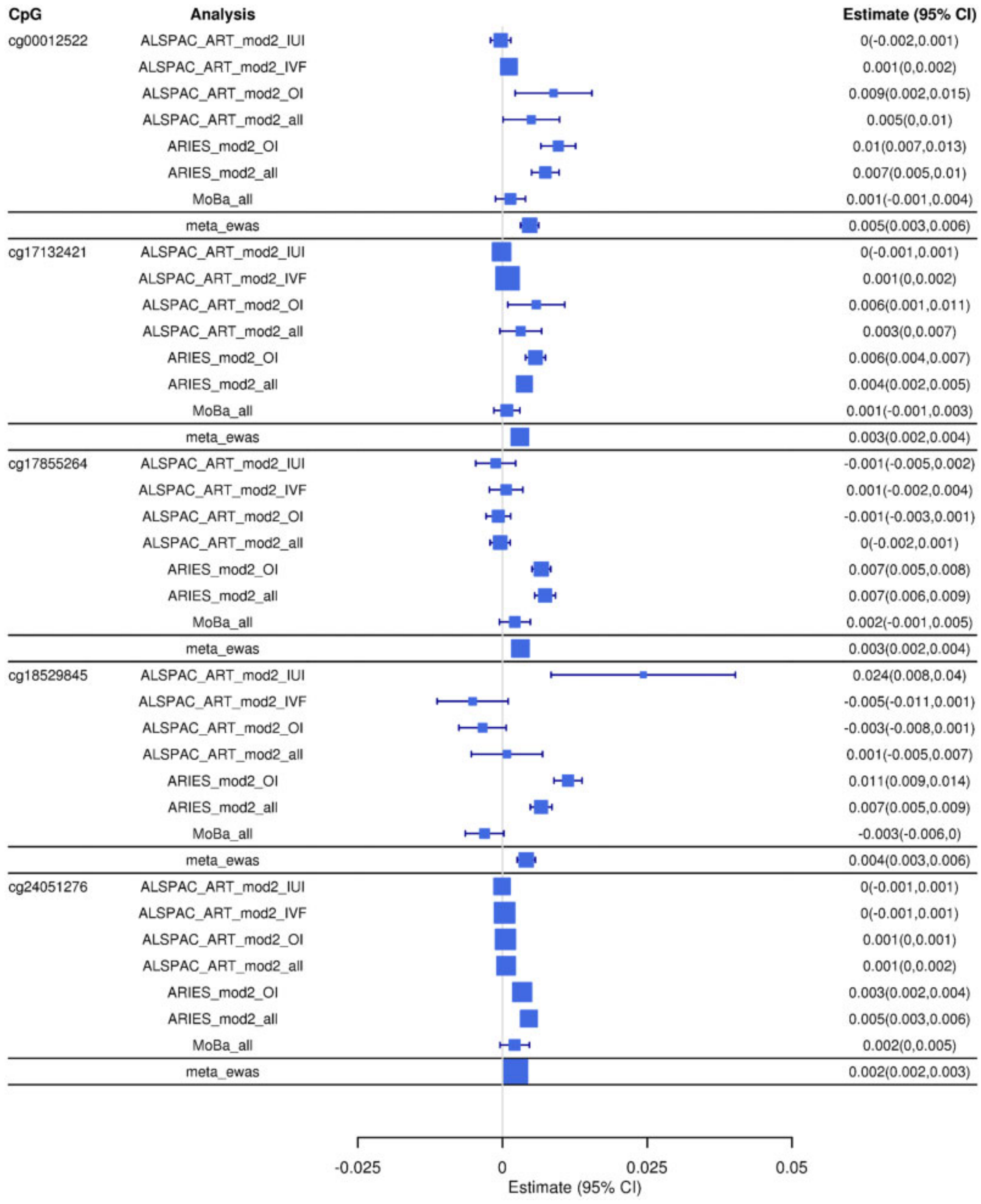

Supplementary Figure S1. Association of cord blood DNA methylation and medically assisted reproduction (beta coefficients and 95% confidence intervals) across the subsamples used in the meta-analysis and by self-reported procedure. ALSPAC-ART, ALSPAC-MAR subsample; ARIES, ALSPAC-ARIES subsample; IUI, intrauterine insemination; OI, ovulation induction; all, medically assisted reproduction.
